# Gonadal Transcriptomic Analysis Reveals Novel Sex-Related Genes in *Bactrocera dorsalis*

**DOI:** 10.3390/insects15060424

**Published:** 2024-06-05

**Authors:** Qin Wang, Yuxuan Lei, Hongjie Lin, Xiaoxin Chen, Wanyu Mo, Boyang Guan, Huimin Deng

**Affiliations:** 1 Guangdong Key Laboratory of Insect Developmental Biology and Applied Technology, South China Normal University, Guangzhou 510631, China; 2022022998@m.scnu.edu.cn (Q.W.); 2023022962@m.scnu.edu.cn (Y.L.); 18613128084@163.com (H.L.); 20222532011@m.scnu.edu.cn (X.C.); 20201132043@m.scnu.edu.cn (W.M.); 20202531029@m.scnu.edu.cn (B.G.); 2 Guangzhou Key Laboratory of Insect Development Regulation and Application Research, Institute of Insect Science and Technology, School of Life Sciences, South China Normal University, Guangzhou 510631, China

**Keywords:** *Bactrocera dorsalis*, transcriptomic annotation, gonads, differential expression, unknown genes

## Abstract

**Simple Summary:**

The oriental fruit fly, *Bactrocera dorsalis*, has become a major threat to fruit and vegetable production due to the serious damage it causes, its worldwide distribution and invasive capacity, and resistance to commonly used insecticides. Moreover, the fly has a high reproductive capacity, and this prolific reproductive capacity can lead to extreme levels of destruction in affected fruits. Considering the fly’s high fertility, targeting the gonads could theoretically be an effective method of pest control. However, the molecular mechanisms underlying gonadal development remain largely unrevealed. To identify gonad-specific expressed genes, an analysis of gonadal transcriptomic sequencing was conducted. A total of 1338, 336, 35, and 479 differentially expressed genes were found in the testis, ovary, female accessory gland, and male accessory gland, respectively. Surprisingly, approximately half of highly expressed gonad-specific genes were uncharacterized. Therefore, the functional motifs or domains of uncharacterized highly expressed gonad-specific genes were predicted, and 23% of novel highly expressed gonad-specific genes encoded proteins that contained signal peptides or transmembrane domains. Furthermore, the spatiotemporal expression and sequence characterization of six novel highly expressed gonad-specific genes were analyzed. Overall, genes associated with gonadal development may aid us in the search for pest control targets to improve pest management approaches.

**Abstract:**

*Bactrocera dorsalis* (Hendel) (Diptera: Tephritidae) is one of the most devastating agricultural pests worldwide due to its high reproductive and invasive abilities. The elucidation of its gonadal developmental characteristics and the identification of sex-related genes will provide a useful genetic basis for reproductive-based pest control. Here, the gonadal transcriptome of *B. dorsalis* was sequenced, and novel gonad-specific expressed genes were analyzed. A total of 1338, 336, 35, and 479 differentially expressed genes (DEGs) were found in the testis (TE), ovary (OV), female accessory gland (FAG), and male accessory gland (MAG), respectively. Furthermore, 463 highly expressed gonad-specific genes were identified, with the TE having the highest number of specific highly expressed genes, at 402, followed by 51 in the OV, 9 in the MAG, and only 1 in the FAG. Strikingly, approximately half of highly expressed gonad-specific genes were uncharacterized. Then, it was found that 35, 17, 3, 2, and 1 of 202 uncharacterized highly expressed TE-specific genes encoded proteins that contained transmembrane domains, signal peptides, high-mobility group boxes, the zinc finger domain, and the BTB/POZ domain, respectively. Interestingly, approximately 40% of uncharacterized highly expressed gonad-specific genes encoding proteins were not predicted to possess functional motifs or domains. Finally, the spatiotemporal expression and sequence characterization of six novel highly expressed gonad-specific genes were analyzed. Altogether, our findings provide a valuable dataset for future functional analyses of sex-related genes and potential target sites for pest control.

## 1. Introduction

The oriental fruit fly, *Bactrocera dorsalis* (Diptera: Tephritidae), is one of the most invasive and destructive agricultural pests worldwide. Originating from the southern regions of India, it has already invaded over 70 countries globally and causes a significant threat to agriculture, international trade, and ecosystems [1,2,3]. *B. dorsalis* mainly causes a decrease in fruit quality and output, as well as increased production costs, due to its larvae feeding on the fruit mass, leading to significant economic losses in fruit-rich regions [4]. It has been estimated that the damage caused by *B. dorsalis* to China’s citrus industry reaches USD 40 billion [5]. 

Existing control methods of *B. dorsalis* include pesticides, biological control, and integrated pest and habitat management. However, although many of these approaches have been highly successful, they also have limitations. For example, the use of pesticides can strongly select for resistance and damage non-pest populations and the environment [6,7]. Thus, there is an urgent need to develop novel, environmentally safe, and efficient technologies for the sustainable control of *B. dorsalis*. The sterile insect technique (SIT) is a method that has been used since the 1950s to control pest populations and involves releasing large numbers of sterile insects into natural populations after mutation. Then, these sterile insects compete for mating with wild insects of the opposite sex, rendering them unable to produce offspring [8]. Therefore, the SIT has been considered a target-specific and environmentally friendly method of pest control. Nowadays, genetics-based inheritable SIT (gSIT) is commonly used. Its principle is to sex-specifically make insects lethal or sterile by knocking out sex-specific genes, thereby controlling pest populations [9,10]. The application of gSIT is based on the elucidation of the principles of reproductive development and the identification of reproduction-related or sex-related genes, especially gonad-specific genes.

Insects’ gonads consist of the testis (TE), ovary (OV), male accessory gland (MAG), and female accessory gland (FAG), each with distinct functions to guarantee high reproductive capacity. Identifying the key factors that regulate gonadal development, mating behavior, and ovulation in *B. dorsalis* would not only facilitate the elucidation of the molecular regulatory mechanism of the insect’s powerful reproductive capacity, but also provide new molecular targets for pest control. Recent research has identified several genes involved in the development of the TE and OV in *B. dorsalis*, such as *Tssk1*, *Tektin1*, *Tudor*, *Nanos*, and *miR-309a* [11,12,13,14]. However, more genes that play important roles in the species’ reproduction require further identification.

Transcriptomic sequencing technology can solve many genetically relevant problems by using high-throughput sequencing technology for analysis to accurately determine the expression levels of specific genes, differential splicing, and allele-specific expression of transcripts. Recently, these advantages have led to its widespread use in transcriptomic profiling studies on gonadal development in various insects, such as *Bombyx mori* [15], *Drosophila melanogaster* [16], *Spodoptera litura* [17], and *Periplaneta americana* [18]. Transcriptomic sequencing has also been used to identify sex-related genes and associated regulatory mechanisms in the TE and OV of *B. dorsalis*. As shown in previous studies, several genes and signal transduction pathways involved in ovarian and testicular development, as well as in sexual determination, have been identified [19,20,21,22]. However, the genes that function in the accessory gland of *B. dorsalis* have not been studied systematically. Additionally, more sex-related genes need to be identified as ideal molecular targets for reproductive-based pest control. 

Here, a comprehensive transcriptomic analysis of mature TE, OV, MAG, and FAG in *B. dorsalis* was performed using second-generation transcriptome-sequencing technology. Firstly, TE-, OV-, MAG-, and FAG-specific genes were screened. Then, highly expressed TE-, OV-, MAG-, and FAG-specific genes were used for functional enrichment. Additionally, the functional prediction of uncharacterized genes that were specifically and highly expressed in the gonads was performed. Finally, six novel gonad-specific highly expressed genes were selected for the spatiotemporal expression pattern analysis. These specific expression data provide new information to elucidate the regulatory mechanisms of gonadal development and sex determination in *B. dorsalis*, helping to develop reproduction-based pest control technologies.

## 2. Materials and Methods

### 2.1. Insects

*B. dorsalis*, provided by the Institute of Plant Protection, Guangdong Academy of Agricultural Sciences, were reared in our laboratory at a temperature of 27 °C, humidity of 70%, and a light–dark cycle of 14 h light and 10 h dark. The growth cycle of *B. dorsalis* is divided into larval, pupal, and adult stages. The larvae were fed with fresh bananas, while the adults were fed with a solid artificial medium. The formulation of the solid medium (1 L) consisted of 90 g sucrose, 30 g yeast extract, 15 g honey, and 10 g agar powder. Adults reached sexual maturity at approximately 9 days after eclosion. 

### 2.2. Sample Collection, RNA Extraction, and Illumina Sequencing

Adults that eclosed on the same day were separated by sex and reared until the 12th day (the sexually mature period) before sampling. After freezing anesthesia, the adults were dissected under a stereomicroscope in cold and sterile phosphate-buffered saline (PBS, pH 7.2) treated with 0.1% diethylpyrocarbonate (DEPC). TE, OV, MAG, and FAG were collected and pooled in the 1.5 mL tubes without RNase. Various gonads from 30 adults were considered as one biological sample, and three independent biological replicates were taken. Then these samples were immediately frozen in liquid nitrogen and stored at −80 °C.

Total RNAs from the various gonads were extracted using Trizol reagent (Invitrogen, Carlsbad, CA, USA) according to the manufacturer’s protocol, and their quantity and purity were examined using a Bioanalyzer 2100 (Agilent, Santa Clara, CA, USA). For single library preparation, the total RNA should be ≥1 μg, concentration ≥ 35 μg/μL, OD260/280 ≥ 1.8, and OD260/230 ≥ 1.0. The Illumina NovaSeq 6000 (Illumina, San Diego, CA, USA) platform was used for sequencing short sequences. The extracted messenger RNA (mRNA) was randomly broken, and small fragments of about 300 bp were separated by magnetic bead selection. These fragments were reverse-transcribed into first-strand complementary DNA (cDNA), and the second-strand was synthesized to form a stable double-stranded structure. The purified double-stranded cDNA was then subjected to terminal repair, poly (A) addition and sequencing splicing, fragment size selection, and PCR amplification. Afterward, the samples were sequenced on the machine to acquire the RNA-Seq data. The raw data have been submitted to the NCBI sequence read archive (SRA) under accession number SRP499373.

### 2.3. Assembly and Sequence Annotation

To ensure the accuracy of the RNA-Seq data and reduce the interference from invalid data, the following methods were used for the quality control of the raw data: The fastp (0.19.5) was used to remove adapters, low-quality sequences, and N bases (below quality three) to obtain high-quality clean reads. Clean reads were mapped to the reference genome of *B. dorsalis* (ASM78921v2, https://www.ncbi.nlm.nih.gov/genome/?term=txid27457[orgn], accessed on 27 June 2022) using HISAT2 (2.1.0). To annotate the functions of unigenes, the mapped reads were annotated with the following databases: non-redundant (NR) databases (ftp://ftp.ncbi.nih.gov/blast/db/, accessed on 27 June 2022), SwissProt (http://www.uniprot.org/, accessed on 27 June 2022), Gene Ontology (GO) (http://www.geneontology.org/, accessed on 11 March 2024), Evolutionary Genealogy of Genes: Non-supervised Orthologous Groups (EggNOG) (http://eggnogdb.embl.de/#/app/emapper, accessed on 9 March 2024), and pfam (http://pfam.xfam.org/, accessed on 14 May 2024) using Blast+ (Version 2.9.0). The above process was performed by Majorbio Bio-Pharm Technology Co., Ltd. (Shanghai, China).

### 2.4. Identification and Functional Annotation of Differentially Expressed Genes

Quantification of gene expression levels was estimated by fragments per kilobase of transcript per million fragments mapped (transcripts per million reads [TPM]) using RSEM (1.3.3). Unigenes with TPM > 1 were considered to be expressed genes. Differentially expressed genes (DEGs) were identified based on a corrected *p*-value (*p*-adjusted) < 0.05 and a fold change (FC) ≥ 2 or FC ≤ 0.5 by using the DEseq2 software (1.24.0). Functional annotations of the DEGs were performed via the analysis of EggNOG enrichment and GO annotations. GO terms with *p*-adjusted < 0.05 were considered to be significantly enriched DEGs.

### 2.5. Functional Analysis of Novel Gonad-Specific Highly Expressed Genes

To investigate the potential function of novel gonad-specific highly expressed genes, the InterProScan online platform (https://www.ebi.ac.uk/interpro/result/InterProScan/, accessed on 14 May 2024) [23] was used to predict the signal peptide (SP), transmembrane (TM) domain, and conserved motifs or domains in the protein coding sequence. Clustal Omega (https://www.ebi.ac.uk/jdispatcher/msa/clustalo, accessed on 25 May 2024) was used for conducting sequence alignments between homologous proteins. 

### 2.6. Quantitative Real-Time Polymerase Chain Reaction Analysis

To determine the spatiotemporal expression patterns of novel gonad-specific highly expressed genes, the samples at different developmental stages and the tissues of mature adults were collected, respectively. Specifically, the 1st, 2nd, and 3rd instar larvae hatched on the same day, and the pupae on the 1st, 5th, and 9th day were collected, respectively. Male and female adults separated by sex after eclosion were collected from the 1st to the 15th day. Three biological replicates were taken for each stage. Collected samples were washed with 0.1% DEPC-treated water, frozen in liquid nitrogen, and then stored at −80 °C for subsequent total RNA extraction. The TE, OV, MAG, FAG, midgut, fat body and epidermis were dissected in 10-day-old adults that were reared separately by sex. Additionally, the remaining tissues were collected as one sample, named “others”. Three biological replicates were taken for each tissue. The collected samples were frozen in liquid nitrogen and stored at −80 °C for subsequent total RNA extraction.

A total of 2 μg RNA extracted from each sample was reverse-transcribed into cDNA using the HI Script III All-in-one RT SuperMix Perfect Kit (Vazyme, Guangzhou, China). Then, a quantitative real-time polymerase chain reaction (qRT-PCR) was performed on an ABI 7500 real-time PCR system (Life Technologies Inc., Carlsbad, CA, USA) using SYBR Green Real-time PCR Master Mix (Vazyme, Biotechnology, Guangzhou, China) according to the following process: denaturation at 95 °C for 30 s, followed by 40 cycles of 95 °C (10 s) and 60 °C (30 s). The *Tubulin alpha-1 chain* gene (Accession number: XM_011212814.3) was used to normalize the expression values. The 2^−ΔCT^ method was used to calculate the relative expression levels of a target gene. The primers used in qRT-PCR are shown in Table S1. All the experiments were carried out at least in triplicate, and the values were expressed as the mean ± S.E.M. The statistical significance of the mRNA expression levels was analyzed using a one-way analysis of variance. 

## 3. Results

### 3.1. Morphological Structure of Mature TE, OV, MAG, and FAG in B. dorsalis

**Figure 1:**
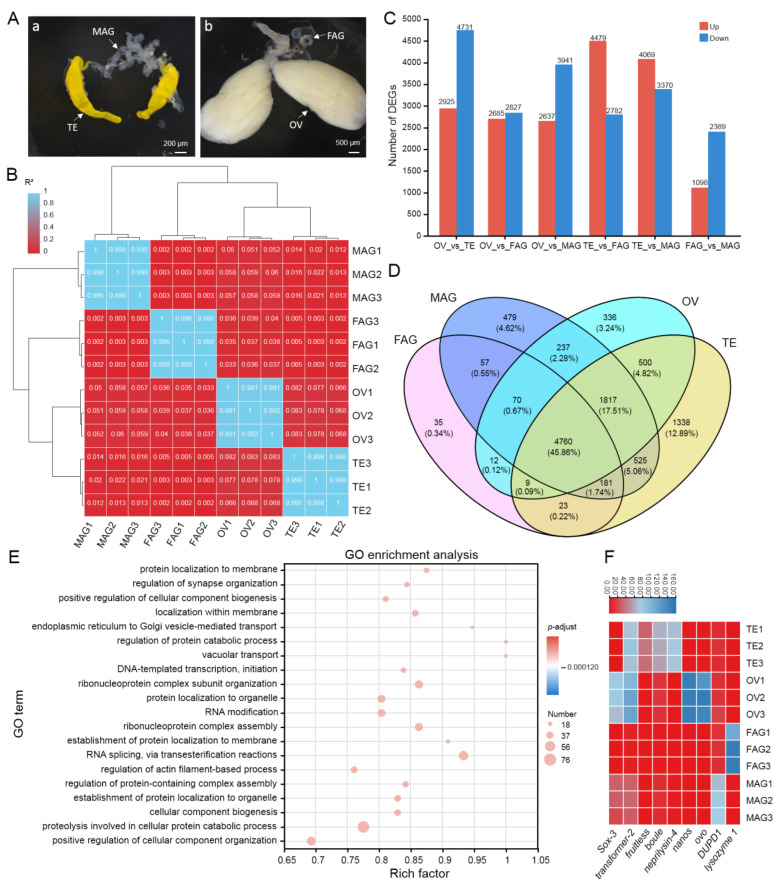
Transcriptomic analysis of four mature gonads in *B. dorsalis*. (**A**) Morphological structures of mature testis (TE), male accessory glands (MAG), ovary (OV), and female accessory glands (FAG) on day 10 of the adult stage. The bar in (a,b) represents 200 µm and 500 µm, respectively. (**B**) Pearson correlation coefficient analysis of TE, MAG, OV, and FAG. (**C**) The number of up- and downregulated DEGs identified in 4 pairwise comparison groups. (**D**) Venn diagram represents the DEGs among TE, MAG, OV, and FAG. (**E**) GO functional enrichment of co-expressed genes in the TE, MAG, OV, and FAG. (**F**) Heatmap of the known sexual determination-related, TE-specific, OV-specific, FAG-specific, and FAG-specific genes.

*B. dorsalis* belongs to holometabolous insects, and its development process consists of embryogenesis, larva, pupa, and adult stages. The gonads are the important organs involved in reproduction, and their development extends from the larval stage to the sexually mature period of the adult. In males, the reproductive glands consist of the TE and MAG, while they comprise the OV and FAG in females. The morphological structure of mature gonads in *B. dorsalis* was observed. The mature TE comprised a pair of elongated and bright yellow sac-like structures with finger-like bends at the top and a twisted base, while the mature MAG consisted of multiple branched transparent tubular structures that intertwined and coiled around each other, and it was attached to the anterior enlarged cup-shaped portion of the ejaculatory duct input segment (Figure 1(Aa)). The pear-shaped OV was covered by a transparent membrane, and it tapered towards the base where it connected to the lateral oviducts, while the FAG comprised a pair of short and thick transparent tubular structures located on both sides at the base of the ovary (Figure 1(Ab)). The TE serves as the primary site for spermatogenesis in insects [20], while the MAG secretes mucus for sperm bathing and preservation [24]. The OV is the primary site for oogenesis [13], while the FAG is generally thought to secrete proteins to promote ovulation and protect eggs [25].

### 3.2. Sequencing Quality Assessment

To identify TE-, OV-, MAG-, and FAG-specific genes, a comparative transcriptomic analysis of the TE, OV, MAG, and FAG in sexually mature flies was performed. A total of 78.11 Gb clean data were obtained from the Illumina sequencing of 12 samples, with each sample’s clean data exceeding 6.11 Gb. The Q30 (the percentage of sequences with a sequencing error rate below 0.1%) base percentages were all above 93.08%, suggesting that the quality of the obtained transcriptomic sequences was high (Table S2). The “Clean Reads” of each sample were aligned to the specified reference genome separately, with alignment rates ranging from 69.86% to 89.07% (Table S3). Specifically, the TE sequencing yielded an average of 45,360,046 raw reads, with an average of 43,709,384 clean reads after quality control, an average Q30 base percentage of 93.23%, an average GC content of 42.93%, and a sequence alignment rate of 80.93% with the reference genome. The OV sequencing yielded an average of 47,228,762 raw reads, with an average of 45,515,266 clean reads after quality control, an average Q30 base percentage of 93.42%, an average GC content of 43.97%, and a sequence alignment rate of 88.96% with the reference genome. The MAG sequencing yielded an average of 45,360,047 raw reads, with an average of 43,709,384 clean reads after quality control, an average Q30 base percentage of 93.23%, an average GC content of 42.93%, and a sequence alignment rate of 70.91% with the reference genome. The FAG sequencing yielded an average of 46,685,763 raw reads, with an average of 44,964,631 clean reads after quality control, an average Q30 base percentage of 94.34%, an average GC content of 45.75%, and a sequence alignment rate of 80.32% with the reference genome. The comparison rate of each sample (Q30) exceeded 93%, suggesting that the RNA sequencing produced high-confidence sequences.

### 3.3. Identification of DEGs in Four Gonads

To investigate the transcriptomic differences among the four gonads, an analysis of Pearson correlation coefficients for the TE, OV, FAG, and MAG was conducted. The similarity values were approximately 0.076, 0.003, 0.016, 0.037, 0.056, and 0.003 between TE and OV, TE and FAG, TE and MAG, OV and FAG, OV and MAG, and FAG and MAG, respectively (Figure 1B), suggesting that the gene expression levels between all the pairs of TE, OV, MAG, and FAG show significant difference. 

To further evaluate the expression levels of the genes, RNA-Seq clean data were mapped to the *B. dorsalis* genome and normalized to TPM. Unigenes with TPM > 1 were considered to be expressed genes. Overall, a total of 9150, 7737, 8124, and 5142 genes were detected in the TE, OV, MAG, and FAG, respectively, of which 4760 (45.86%) genes were co-expressed in the four gonads (Figure 1D). Gonadal co-expressed genes were enriched into 710 GO items (*p*-adjusted < 0.05), of which the top five were protein localization to membrane, regulation of synapse organization, positive regulation of cellular component biogenesis, localization within membrane, and endoplasmic reticulum to Golgi vesicle-mediated transport (Figure 1E), suggesting that the gonads require a large number of synthesis and transport biomolecules to facilitate gamete maturation and subsequent mating and ovulation. Based on the criteria of fold change ≥ 2 and *p*-adjusted < 0.05, 7656, 7261, 7439, 5512, 6578, and 3487 DEGs were identified between TE and OV, TE and FAG, TE and MAG, OV and FAG, OV and MAG, and FAG and MAG, respectively (Figure 1C). Furthermore, a total of 10,836 DEGs between all pairs of the TE, OV, MAG, and FAG were identified, among which 1338, 336, 35, and 479 DEGs were specifically expressed in the TE, OV, FAG, and MAG, respectively (Figure 1C,D), revealing that the DEGs might contribute to the functional diversity of the four gonads, and that the TE has the highest number of DEGs compared to the other gonads. 

To test the accuracy of the DEG analysis, some usual candidate genes specifically expressed in the TE, OV, MAG, and FAG were searched in the transcriptome. *Sox3*, a female sexual determination gene [26], shows high expression in the OV of *B*. *dorsalis*. *Transformer2*, the key factor of the insect sex-determination system [27], exhibits high mRNA expression levels both in the TE and OV, but its expression in the OV is higher than in the TE. *Fruitless*, a male sexual determination gene [28], is only expressed in the TE. Additionally, the mRNA of *boule* and *neprilysin-4,* important for spermatogenesis [29,30], *nanos* and *ovo*, essential for oogenesis [31,32], *dupd1*, the encoding products of which belong to the seminal fluid peptides [33], and *lysozyme 1,* related to the antimicrobials of FAG [34], are specifically expressed in the TE, OV, MAG, and FAG, respectively (Figure 1E). This suggests that the DEG analysis is relatively accurate. 

### 3.4. Analysis of Gonad-Specific Highly Expressed Genes 

**Figure 2:**
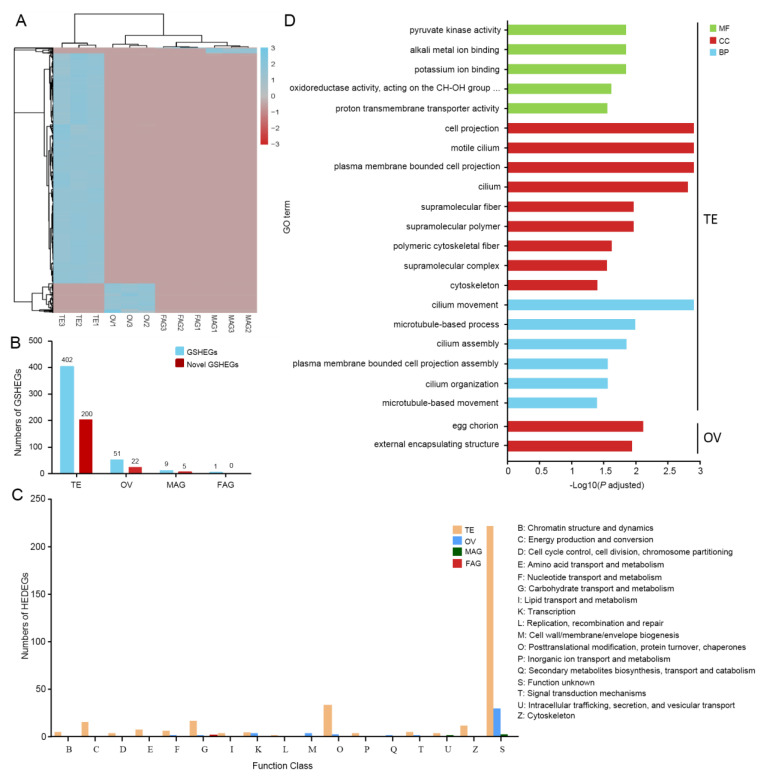
Analysis of gonad-specific highly expressed genes. (**A**) Heatmap and hierarchical clustering of the gonad-specific highly expressed genes (GSHEGs). The genes with a TPM > 100 in one gonad and TPM < 1 in the other gonads were referred to as gonad-specific highly expressed. (**B**) Number of GSHEGs and novel GSHEGs in the TE, OV, MAG, and FAG. (**C**) The eggNOG functional classification of GSHEGs in the TE, OV, MAG, and FAG. (**D**) GO annotated analysis of TE- and OV-specific highly expressed genes. MF, molecular function. CC, cellular component. BP, biological process.

The root of different functions arising in different tissues lies in differential gene expression, particularly the genes that are highly expressed in a tissue-specific manner, which are often essential for tissue-specific functions [35]. Therefore, screening of the specific highly expressed genes in TE, OV, MAG, and FAG was conducted based on the standard with TPM > 100 in a gonad and the TPM < 1 in the other gonads. A total of 463 gonad-specific highly expressed genes were identified, with the TE having the most specific highly expressed genes at 402, followed by 51 in the OV, 9 in the MAG, and only 1 in the FAG (Table S4 and Figure 2A,B). 

Furthermore, the functional prediction and classification of gonad-specific highly expressed genes were conducted by searching the EggNOG and GO databases. Among 402 TE-specific highly expressed genes, 202 genes were annotated and classified into 15 functional categories, the top 3 of which were: O category “Posttranslational modification, protein turnover, chaperones” (33 genes, 7.13%), G category “Carbohydrate transport and metabolism” (16 genes, 3.45%), and C category “Energy production and conversion” (15 genes, 3.24%) (Figure 2B,C). Unexpectedly, 200 TE-specific highly expressed genes have no functional annotation in the EggNOG database, accounting for 49.75% (Figure 2B). Among 51 ovary-specific highly expressed genes, 29 genes were annotated and classified into 15 functional categories, the top 3 of which were K category “Transcription” (3 genes, 5.88%), M category “Cell wall/membrane/envelope biogenesis” (3 genes, 5.88%), and O category “Posttranslational modification, protein turnover, chaperones” (2 genes, 3.92%) (Figure 2B,C). In addition, 22 out of the 51 ovary-specific highly expressed genes were unannotated (Figure 2B). Among the nine MAG-specific highly expressed genes, only one gene (*LOC105223294*) was enriched in the U category “Intracellular trafficking, secretion, and vesicular transport” (Figure 2B,C). The FAG-specific highly expressed gene (*LOC105228345*) was enriched in the G category “Carbohydrate transport and metabolism” (Figure 2C). These results reveal that approximately half of the gonad-specific highly expressed genes are functionally uncharacterized, being considered novel genes. Then, the TE-specific highly expressed genes were classified into three major GO categories based on the GO enrichment (Figure 2D). Among these functional groups, the terms “cell projection”, “motile cilium”, “plasma membrane bounded cell projection”, and “cilium” were dominant in the cellular component categories, while the term “cilium movement” was dominant in the biological process categories (*p*-adjusted < 0.05) (Figure 2D), associated with sperm maturation and motility. The OV-specific highly expressed genes were significantly enriched in the cellular component categories, including “external encapsulating structure” and “egg chorion” (Figure 2D), important for follicle development and egg maturation. Due to a small number of MAG/FAG-specific highly expressed genes, no related functional categories were enriched (Figure 2D). These data suggest that the gonad-specific highly expressed genes are mainly related to gamete maturation. In addition, the numerous uncharacterized gonad-specific highly expressed genes indicate that the molecular regulatory mechanism of reproduction in *B. dorsalis* remains largely unrevealed.

### 3.5. Functional Prediction of Novel Gonad-Specific Highly Expressed Genes

**Figure 3:**
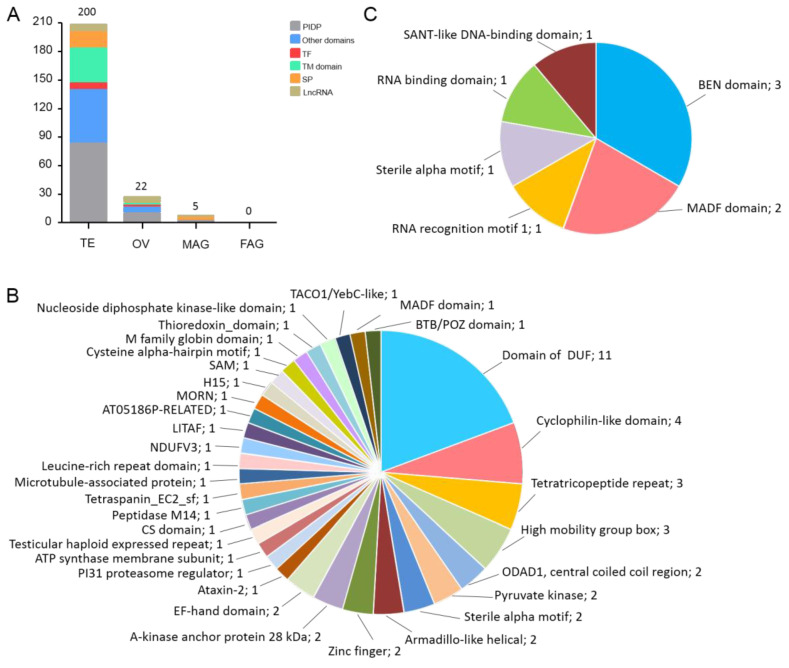
Functional prediction of novel gonad-specific highly expressed genes. (**A**) Classification of novel gonad-specific highly expressed genes in the TE, OV, MAG, and FAG based on the predicted functional motifs or domains of their encoding proteins. PIDP, potential intrinsically disordered protein. TF, transcription factor. TM domain, transmembrane domain. SP, signal peptide. LncRNA, long non-coding RNA. (**B**) Predicted functional motifs or domains of novel TE-specific highly expressed genes encoding proteins. (**C**) Predicted functional motif or domain of novel OV-specific highly expressed genes encoding proteins.

It was noted that approximately half of gonad-specific highly expressed genes were uncharacterized genes. To further investigate the functions of these uncharacterized genes, motifs or domains, the functional structural components of proteins, were predicted in the novel gonad-specific highly expressed genes encoding proteins. Out of the 202 novel TE-specific highly expressed genes, 194 were predicted protein-encoding genes (Figure 3A), among which 35 and 17 contained a signal peptide and a transmembrane domain, respectively (Figure 3A). In addition, eight novel TE-specific highly expressed genes encoded transcription factors, possessing a high mobility group (HMG) box (three), zinc finger domain (two), BTB/POZ domain (one), TACO1/YebC-like domain (one) and MADF domain (one), respectively. Then, 49 novel TE-specific highly expressed genes encoding proteins were predicted to have other functional domains, such as a DUF domain (11), tetratricopeptide repeat (3), central coiled coil region (2), ATP synthase membrane subunit K (1), pyruvate kinase (2), sterile alpha motif (2), armadillo-like helical (2) and cysteine alpha-hairpin motif (1). Additionally, no functional motifs or domains were predicted in the 85 novel TE-specific highly expressed genes encoding proteins, which are probably intrinsically disordered proteins (IDPs) (Figure 3A,B). Among the 22 novel OV-specific highly expressed genes, there were 16 protein-encoding genes, 1 of which contained a TM domain, and 2 of which were transcription factors with a MADF domain and Myb/SANT-like DNA-binding domain, respectively (Figure 3A,C). Furthermore, seven genes encoded other functional proteins, three of which contained a BEN domain, one of which contained a sterile alpha motif, and one of which contained an RNA-binding domain (Figure 3C). Additionally, six OV-specific highly expressed genes encoding proteins belonged to potential IDPs (Figure 3A). Among the novel MAG-specific highly expressed gene set, three genes encoded proteins with SP (Figure 3A). Approximately 40% and 26% of novel gonad-specific highly expressed genes encoding proteins belong to potential IDPs and transporter proteins, respectively, indicating that there is active physiological activity in the mature gonads, in preparation for gamete maturation and mating. 

### 3.6. The Gonad-Specific Transcript Profiling of Six Selected Genes

To investigate the specific functions of uncharacterized genes in the gonads, the spatiotemporal expression patterns of two novel OV-specific genes (*LOC105224822* and *LOC105229677*), two novel TE-specific genes (*LOC105225947* and *LOC105227624*), one novel FAG-specific gene (*LOC105228345*), and one novel MAG-specific gene (*LOC105227112*) were explored using qRT-PCR, respectively. 

**Figure 4:**
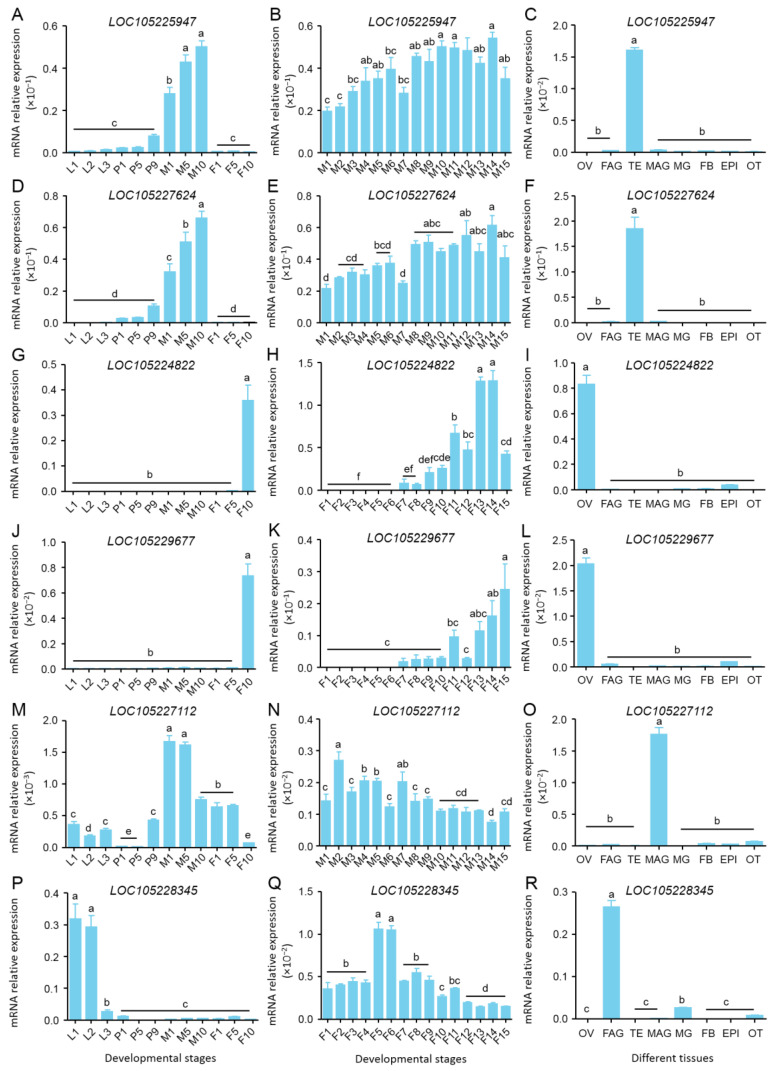
The gonad-specific transcript profiling of 6 selected genes detected by qRT-PCR. (**A**–**C**) Spatiotemporal expression pattern of novel TE-specific highly expressed gene *LOC105225947*. (**D**–**F**) Spatiotemporal expression pattern of novel TE-specific highly expressed gene *LOC105227624*. (**G**–**I**) Spatiotemporal expression pattern of novel OV-specific highly expressed gene *LOC105224822*. (**J**–**L**) Spatiotemporal expression pattern of novel OV-specific highly expressed gene *LOC105229677*. (**M**–**O**) Spatiotemporal expression pattern of novel MAG-specific highly expressed gene *LOC105227112*. (**P**–**R**) Spatiotemporal expression pattern of novel FAG-specific highly expressed gene *LOC105228345*. Different letters above the bars indicate significant differences (least significant difference in one-way analysis of variance, *p* < 0.05). Ln, stage of larval instar. Pn, day of pupal stage. Mn, day of male adults. Fn, day of female adults. MG, midgut. FB, fat body. EPI, epidermis. OT, remain tissues. OV, ovary. TE, testis. FAG, female accessory gland. MAG, male accessory gland.

The mRNA expression levels of *LOC105225947* and *LOC105227624* were both undetectable in the larvae and female adults, relatively low in the pupae, and high in the male adults (Figure 4A,D). Moreover, their mRNA expression levels remained high during the male adult stage (Figure 4B,E). Then, the detection of tissue-specific expression levels showed that they were highly expressed in the TE of adults on day 10, but at an undetectable level in other tissues (Figure 4C,F), which is consistent with the results of RNA-Seq. Therefore, it was speculated that *LOC105225947* and *LOC105227624*, the male-related genes, may play roles in spermatogenesis, sperm maturation, and mating.

The mRNA expressions of *LOC105224822* and *LOC105229677* were both at undetectable levels in the larvae, pupae, and male adults, while they showed high levels in the female adults (Figure 4G,J), suggesting that they are female-related genes. In addition, their mRNA expression levels were both low in early female adults, gradually increased until day 7 of the female adult stage, and then reached high levels at the sexual maturity stage (Figure 4H,K). As expected, they showed OV-specific expressions in adults on day 10 (Figure 4I,L). Thus, the results imply that *LOC105224822* and *LOC105229677*, the female-related genes, may have functions in oocyte maturation and ovulation.

Although the mRNA expression levels of *LOC105227112* and *LOC105228345* showed MAG-specific and FAG-specific expressions on day 10 in adults, respectively (Figure 4O,R), they also had relatively high expressions in the whole larvae (Figure 4M,P), indicating that they may function in both early and late development of accessory glands. 

The above results not only verify the accuracy of the RNA-seq results that the identified genes are gonad-specific expression genes in mature adult of *B. dorsalis*, but also indicate their potential functions in reproduction. 

### 3.7. Sequence Characterization of Six Selected Sex-Related Genes

**Figure 5:**
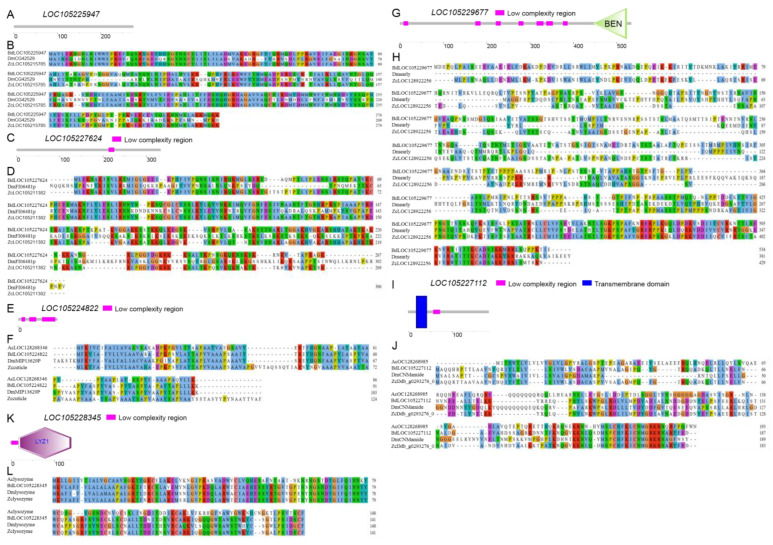
Sequence characterization of six selected sex-related genes. (**A**,**C**,**E**,**G**,**I**,**K**) Functional domain prediction of LOC105225947, LOC105227624 LOC105224822, LOC105229677, LOC105227112, and LOC105228345, respectively. (**B**,**D**,**F**,**H**,**J**,**L**) Comparison of the amino acid sequences of LOC105225947, LOC105227624 LOC105224822, LOC105229677, LOC105227112, and LOC105228345 of *B. dorsalis* with other insects. Bd, *B. dorsalis*. Zc, *Zeugodacus cucurbitae*. Dm, *D. melanogaster*. Ac, *Anopheles cruzii*. Regions with the same color represents conserved sites of the sequence. Hydrophobic amino acid residues are colored blue if the column includes more than 60% hydrophobic amino acid residues. Positive charge amino acid residue K or R is colored red if the column includes more than 60% K and R, or more than 80% of either K or R. Negative charge amino acid residue E or D is colored magenta if the column includes more than 50% E and D, or more than 85% of either E or D. Polar amino acid residue N, Q, S or T is colored green if the column includes more than 60% N, or more than 85% Q, or more than 85% of either S or T. Cysteine is colored pink if the the column includes more than 85% C.

To further elucidate the structural characteristics, the sequence features of the above six gonad-specific genes were investigated. The prediction results show that the LOC105224822, LOC105227112, and LOC105228345 proteins have signal peptides, respectively, while the LOC105224822, LOC105227112, and LOC105228345 proteins have transmembrane domains, respectively (Table 1). The results of functional domain prediction indicate that the LOC105225947 protein has no functional domain (Figure 5A), the LOC105227624 protein contains one low complexity region (LCR) (Figure 5C), the LOC105224822 protein contains three LCRs (Figure 5E), the LOC105229677 protein contains the BEN functional domain located at the amino acid sites 445 to 523 and seven LCRs (Figure 5G), the LOC105227112 protein contains a transmembrane domain located at amino acid sites 20 to 42 and one LCR (Figure 5I), and the LOC105228345 protein contains a LYZ1 functional domain located at amino acid sites 20 to 141 and one LCR (Figure 5K).

Amino acid sequence alignment and analysis showed that the amino acid sequences of LOC105225947 are conserved in Tephridae and Drosophilidae (Figure 5B); those of LOC105227624 are conserved in Tephridae and Drosophilidae, with highly conserved regions mainly at the N-terminal (Figure 5D); those of LOC105224822 are conserved in Tephridae, Drosophilidae, and Culicidae (Figure 5F); those of LOC105229677 are conserved in Tephridae and Drosophilidae, with highly conserved regions mainly at the C-terminal (Figure 5H); those of LOC105227112 are conserved in Tephridae, Drosophilidae, and Culicidae, with highly conserved regions mainly at the C-terminal (Figure 5J); and those of LOC105228345 are conserved in Tephridae, Drosophilidae, and Culicidae (Figure 5L).

Taken together, the above results reveal the potential functions of six selected novel genes in the reproduction of *B. dorsalis* and their conservation in insects. 

## 4. Discussion

*B. dorsalis* is a significant pest, with a global distribution and rapid reproduction, and it causes substantial damage to fruit and vegetable production [36]. Recently, the SIT has been applied to the control of pests such as *Aedes aegypti* and *Ceratitis capitata* [37,38]. The successful application of the SIT is based on the elucidation of the reproductive developmental rules and the identification of reproduction-related or sex-related genes, especially the gonad-specific genes. Thus, it is important to identify the gonad-specific expressed or sex-related genes. Here, we report the novel gonad-specific highly expressed genes in *B. dorsalis* based on a transcriptomic sequencing analysis of four gonads and the spatiotemporal expression patterns and structural characteristics of six selected genes.

Identification of DEGs from the gonadal transcriptome is an effective method of investigating the molecular differences that regulate gonadal development, sexual differentiation, and sexual determination [39]. In this study, a cDNA library of the TE, OV, MAG, and FAG of *B. dorsalis* was constructed for the first time, with Q30 > 93.0% of the sequences, suggesting the high quality of the data obtained. Then, a comparative transcriptomic data analysis showed that the abundance of male-related genes was much greater than that of the female-related genes, with the TE possessing the highest number of DEGs (1338), followed by MAG (479), OV (338), and FAG (35) (Figure 1C,D). Furthermore, the TE had the most gonad-specific highly expressed genes (402), followed by the OV (51), MAG (9), and FAG (1) based on the standard with TPM > 100 in a specific gonad and TPM < 1 in the other gonads. (Figure 2A,B). Recent reports have also shown that the number of male-related genes from the mature gonads is much larger than that of female-related genes in other species such as *Bombyx mori* [40], *Henosepilachna vigintioctopunctata* [41], green mud crab *Scylla paramamosain* [42], red-tail catfish *Mystus wyckioides* [43], paradise fish *Macropodus opercularis* [44], and *Hemibarbus maculatus* [45]. This indicates that despite the gene expression profiles varying between species, the overall expression patterns for sex-related genes may be conserved across metazoan species. Additionally, this is consistent with the “male sex drive” hypothesis, which states that female development is a “default” state; the direct or indirect activation of male-related genes and the suppression of ovarian-development-related genes during male development are key to genetic variation between the sexes [46,47].

Surprisingly, a large number of uncharacterized gonad-specific highly expressed genes were identified in *B. dorsalis* (Figure 2B), and then qRT-PCR confirmed the gonad-specific expression of the selected genes (Figure 4). A recent ovary-specific transcriptome analysis of *B. dorsalis* showed that 64.95% of unigenes have significant matches in the Nr database and only 46.4% of the total sequences are classified into GO annotated items [16]. In addition, amongst the 30,516 unigenes, almost a third shared no significant similarities with known genes and may be novel/or fast-evolving sequences [20]. The numerous uncharacterized genes found in the gonadal transcriptome of *B. dorsalis* suggest that the molecular mechanisms of gonadal development, sexual differentiation, and sexual determination remain largely unknown.

To further investigate the molecular mechanisms underlying the gonadal development, sexual differentiation, and sexual determination of *B. dorsalis*, we focused on the uncharacterized gonad-specific highly expressed genes. The conserved functional motifs or domains were predicted, and approximately 23% of the novel gonad-specific highly expressed genes encoding proteins contained SP or TM domains (Figure 3A). It was not surprising that many novel gonad-specific highly expressed genes were associated with metabolic processes and TM transport, indicating that the highly coordinated physiological processes of nutrient storage and energy acquisition occur during gamete maturation and subsequent mating. Moreover, this also suggests that the regulation of nutrient storage and energy acquisition in sperm and oocyte maturation might be gonad-specific. In addition, three novel TE-specific highly expressed genes encoding proteins contained an HMG box (Figure 3B). The SOX family genes are a series of SOX transcription factors with a DNA-binding HMG box domain, and are the main source of sex-determining genes or candidate genes. Sry (Sex-Determining region of Y chromosome) is the first SOX transcription factor to be discovered and is involved in male sex determination in mammals [48]. In reptiles, *Sox9* is involved in male sexual differentiation downstream of the male sex-determining gene *Dmrt1* [49,50]. *D. melanogaster SoxE* is important for testis development [51]. Therefore, the identification of novel SOX transcription factors in *B. dorsalis* testis might reveal a new molecular mechanism of male sexual differentiation or determination in insects. Additionally, one and two novel TE-specific highly expressed genes encoding proteins contained a BTB/POZ domain and a zinc finger, respectively (Figure 3B). *Fruitless*, encoding a BTB-zinc-finger transcription factor, is a key sex-determining gene [52]. Furthermore, two novel OV-specific highly expressed genes possessed RNA recognition motif 1 or an RNA binding motif (Figure 3C). *Transformer-2* plays an essential role in the sex-specific splicing of *doublesex* and *fruitless*, and it contains a conserved RNA recognition motif [53]. Thus, these novel TE- and OV-specific highly expressed genes in *B. dorsalis* provide a reliable basis for novel sex-related genes. 

Interestingly, approximately 40% of the novel gonad-specific highly expressed genes in *B. dorsalis* did not have functional motifs or domains (Figure 3A), probably belonging to IDPs. IDPs or their segments do not usually have structure domains under physiological conditions; instead, they adopt multiple interconverting conformational states to adapt to various physiological states [54,55]. A large number of potential IDPs in the gonads of *B. dorsalis* might be one of the reasons that the insects have a strong adaptability and fertility.

In summary, RNA-seq was used to screen novel gonad-specific highly expressed genes, and their gonad-specific expressions were confirmed by qRT-PCR. A total of 205 DEGs might be novel male-related genes, while 22 DEGs might be novel female-related genes. These results provide insight into the molecular mechanisms of gonadal development in *B. dorsalis*, as well as a useful genetic basis for reproductive-based pest control for producing sterile populations.

## Figures and Tables

**Table 1:** Sequence characterization of six selected genes.

Gene ID	Signal Peptide	Transmembrane Domain
LOC105225947	−	−
LOC105227624	−	−
LOC105224822	+	+
LOC105229677	−	−
LOC105227112	+	+
LOC105228345	+	+

The + indicates that the signal peptide or transmembrane domain is predicted, while the − indicates that no signal peptide or transmembrane domain is predicted.

## Data Availability

All the data needed to evaluate the conclusions in this paper are presented in the paper and the Supplementary Materials. Transcriptome reads were deposited in the Sequence Read Archive (SRA) database of NCBI with the accession number SRP499373. Additional data related to this paper can be requested from the authors.

## Supplementary Materials

The following supporting information can be downloaded at https://www.mdpi.com/article/10.3390/insects15060424/s1, Table S1. Primers used in in this study; Table S2. Transcriptomic sequencing data statistics; Table S3 Statistics of the read assignment to the reference genome; Table S4. Gonad-specific highly expressed genes.
