# Supplementary material for: Gonadal Transcriptomic Analysis Reveals Novel Sex-Related Genes in Bactrocera dorsalis
Source: Insects. 2024 Jun 5;15(6):424. doi: 10.3390/insects15060424 (PMC11203884; doi:10.3390/insects15060424)
Supplement: Supplementary file 1 [file insects-15-00424-s001.zip › insects-2988021-supplementary.pdf]

Supplementary Information

Table S1. Primers used in in this study

Primer	Forward (5'-3')	Reverse (5'-3')
LOC105225947	CACGCCCATACTACCGAGTC	GGCCAGGATGAGCGTGATTAG
LOC105227624	TAGCAAAGTCTCGCGTCCAG	GGACTTCTTGCGCTTACCGT
LOC105224822	TGCGCATTACTCGGTCATCA	TGGACATGGTAGTGTCGGGA
LOC105229677	TTTACCGGCCCATTACCCG	AGTCGTCAGTAGGGGGCAAG
LOC105227112	TTCGAGGAGCGTCTAAAGCA	AGTGGCAAGGTGACATATCTGATT
LOC105228345	AACATTCACCCGTTGCTCCT	ACCGTTAGAGTTGGTTGGGC
α-Tubulin	TGCGCATTCATGGTTGATAACG	TAGGGCACCAAGTTAGTCTGGA

Table S2. Transcriptomic sequencing data statistics

Sample	Raw reads	Raw bases	Clean reads	Clean bases	Error rate (%)	Q20 (%)	Q30 (%)	GC content (%)
MAG3	44280504	6686356104	42587640	6162748639	0.0262	97.59	93.18	43
MAG2	45363392	6849872192	43755606	6323267262	0.0262	97.57	93.12	42.96
MAG1	46436244	7011872844	44784906	6440505074	0.026	97.67	93.38	42.82
TE3	49468872	7469799672	47839540	6897982486	0.0259	97.69	93.38	44.73
TE2	49169674	7424620774	47605434	6883096251	0.0261	97.61	93.18	45.03
TE1	44759762	6758724062	43337820	6291085671	0.0254	97.9	93.88	44.85
FAG3	44452088	6712265288	42824422	6109517486	0.0253	97.92	93.97	45.76
FAG2	48504670	7324205170	46497008	6594799354	0.0247	98.15	94.61	45.66
FAG1	47100530	7112180030	45572462	6496874975	0.0249	98.09	94.43	45.82
OV3	49907630	7536052130	48229530	7065880054	0.0257	97.76	93.58	43.98
OV2	44906474	6780877574	43771970	6369484467	0.0262	97.58	93.08	43.94
OV1	46872184	7077699784	44544298	6479608156	0.0257	97.77	93.6	43.99

Table S3 Statistics of the read assignment to the reference genome

Sample	Total reads	Total mapped	Multiple mapped	Uniquely mapped
MAG3	42587640	30536068 (71.7%)	324229 (0.76%)	30211839 (70.94%)
MAG2	43755606	31142219 (71.17%)	313313 (0.72%)	30828906 (70.46%)
MAG1	44784906	31285399 (69.86%)	354770 (0.79%)	30930629 (69.06%)
TE3	47839540	38704251 (80.9%)	194435 (0.41%)	38509816 (80.5%)
TE2	47605434	38491658 (80.86%)	199863 (0.42%)	38291795 (80.44%)
TE1	43337820	35123010 (81.04%)	179730 (0.41%)	34943280 (80.63%)
FAG3	42824422	34543278 (80.66%)	3586686 (8.38%)	30956592 (72.29%)
FAG2	46497008	36921720 (79.41%)	4238048 (9.11%)	32683672 (70.29%)
FAG1	45572462	36860422 (80.88%)	4339297 (9.52%)	32521125 (71.36%)
OV3	48229530	42959771 (89.07%)	285621 (0.59%)	42674150 (88.48%)
OV2	43771970	38879296 (88.82%)	214007 (0.49%)	38665289 (88.33%)
OV1	44544298	39643835 (89.0%)	295548 (0.66%)	39348287 (88.34%)

Table S4. Gonad-specific highly expressed genes

TE	OV	MAG	FAG
LOC105221798	*LOC105221873*	*LOC105223294*	*LOC105228345*
LOC105221844	*LOC105222779*	*LOC105223525*	
LOC105221845	*LOC105222940*	*LOC105223677*	
LOC105221852	*LOC105223468*	*LOC105227112*	
LOC105221857	*LOC105223555*	*LOC105229217*	
LOC105221883	*LOC105223606*	*LOC105231782*	
LOC105221972	*LOC105223627*	*LOC115065996*	
LOC105222015	*LOC105223774*	*LOC115066194*	
LOC105222017	*LOC105223848*	*LOC115066389*	
LOC105222019	*LOC105223978*		
LOC105222020	*LOC105224009*		
LOC105222021	*LOC105225105*		
LOC105222044	*LOC105225218*		
LOC105222079	*LOC105225327*		
LOC105222082	*LOC105226609*		
LOC105222150	*LOC105226624*		
LOC105222164	*LOC105227814*		
LOC105222360	*LOC105227930*		
LOC105222385	*LOC105228039*		
LOC105222389	*LOC105229122*		
LOC105222413	*LOC105229676*		
LOC105222423	*LOC105229677*		
LOC105222432	*LOC105229740*		
LOC105222473	*LOC105229744*		
LOC105222501	*LOC105229944*		
LOC105222502	*LOC105230057*		
LOC105222507	*LOC105230264*		
LOC105222534	*LOC105230676*		
LOC105222588	*LOC105231189*		
LOC105222707	*LOC105231889*		
LOC105222850	*LOC105231955*		
LOC105222924	*LOC105232261*		
LOC105222968	*LOC105232499*		
LOC105223009	*LOC105232507*		
LOC105223023	*LOC105232541*		
LOC105223062	*LOC105232991*		
LOC105223166	*LOC105232993*		
LOC105223180	*LOC105233226*		
LOC105223213	*LOC105233247*		
LOC105223233	*LOC105233902*		

LOC105223339	*LOC105233962*
LOC105223347	*LOC105234029*
LOC105223349	*LOC105234202*
LOC105223350	*LOC109579523*
LOC105223363	*LOC109579646*
LOC105223368	*LOC109579750*
LOC105223402	*LOC115065843*
LOC105223419	*LOC115066280*
LOC105223422	*LOC115066357*
LOC105223490	*LOC115066414*
LOC105223502	*LOC115066422*
LOC105223514	
LOC105223523	
LOC105223553	
LOC105223605	
LOC105223664	
LOC105223688	
LOC105223692	
LOC105223696	
LOC105223790	
LOC105223800	
LOC105223834	
LOC105223847	
LOC105223885	
LOC105223887	
LOC105223922	
LOC105223934	
LOC105223976	
LOC105224084	
LOC105224086	
LOC105224189	
LOC105224220	
LOC105224288	
LOC105224294	
LOC105224322	
LOC105224356	
LOC105224357	
LOC105224384	
LOC105224420	
LOC105224510	
LOC105224567	
LOC105224574	

LOC105224584
LOC105224596
LOC105224626
LOC105224642
LOC105224645
LOC105224646
LOC105224681
LOC105224684
LOC105224708
LOC105224718
LOC105224736
LOC105224737
LOC105224767
LOC105224792
LOC105224851
LOC105224867
LOC105224877
LOC105224908
LOC105224913
LOC105224916
LOC105224975
LOC105224978
LOC105225007
LOC105225052
LOC105225065
LOC105225082
LOC105225084
LOC105225085
LOC105225109
LOC105225151
LOC105225220
LOC105225236
LOC105225253
LOC105225302
LOC105225425
LOC105225444
LOC105225598
LOC105225621
LOC105225656
LOC105225663
LOC105225704
LOC105225723

LOC105225945
LOC105225947
LOC105225963
LOC105225965
LOC105226125
LOC105226134
LOC105226140
LOC105226147
LOC105226204
LOC105226234
LOC105226301
LOC105226304
LOC105226330
LOC105226409
LOC105226460
LOC105226473
LOC105226652
LOC105226653
LOC105226654
LOC105226685
LOC105226772
LOC105226780
LOC105226873
LOC105226911
LOC105226941
LOC105226948
LOC105227068
LOC105227077
LOC105227079
LOC105227120
LOC105227201
LOC105227234
LOC105227242
LOC105227313
LOC105227316
LOC105227417
LOC105227429
LOC105227515
LOC105227529
LOC105227624
LOC105227687
LOC105227689

LOC105227707
LOC105227716
LOC105227738
LOC105227758
LOC105227788
LOC105227817
LOC105227890
LOC105227891
LOC105227945
LOC105227955
LOC105227968
LOC105227969
LOC105227971
LOC105227972
LOC105228008
LOC105228020
LOC105228022
LOC105228024
LOC105228062
LOC105228068
LOC105228098
LOC105228177
LOC105228224
LOC105228344
LOC105228431
LOC105228453
LOC105228454
LOC105228501
LOC105228502
LOC105228526
LOC105228552
LOC105228554
LOC105228556
LOC105228560
LOC105228561
LOC105228562
LOC105228576
LOC105228599
LOC105228647
LOC105228648
LOC105228687
LOC105228707

LOC105228709
LOC105228754
LOC105228798
LOC105228894
LOC105228900
LOC105228931
LOC105228973
LOC105229018
LOC105229023
LOC105229116
LOC105229143
LOC105229160
LOC105229161
LOC105229171
LOC105229175
LOC105229206
LOC105229293
LOC105229336
LOC105229341
LOC105229352
LOC105229416
LOC105229422
LOC105229423
LOC105229425
LOC105229489
LOC105229499
LOC105229501
LOC105229507
LOC105229516
LOC105229580
LOC105229603
LOC105229648
LOC105229707
LOC105229766
LOC105229774
LOC105229806
LOC105229827
LOC105229835
LOC105229837
LOC105229916
LOC105229919
LOC105229920

LOC105229983
LOC105230138
LOC105230139
LOC105230143
LOC105230144
LOC105230158
LOC105230176
LOC105230180
LOC105230229
LOC105230240
LOC105230309
LOC105230310
LOC105230311
LOC105230387
LOC105230402
LOC105230404
LOC105230413
LOC105230471
LOC105230476
LOC105230477
LOC105230481
LOC105230552
LOC105230562
LOC105230580
LOC105230600
LOC105230644
LOC105230645
LOC105230648
LOC105230664
LOC105230696
LOC105230741
LOC105230743
LOC105230769
LOC105230795
LOC105230817
LOC105230842
LOC105230859
LOC105231017
LOC105231018
LOC105231066
LOC105231182
LOC105231186

LOC105231220
LOC105231223
LOC105231224
LOC105231307
LOC105231311
LOC105231319
LOC105231338
LOC105231361
LOC105231406
LOC105231424
LOC105231431
LOC105231602
LOC105231654
LOC105231713
LOC105231722
LOC105231726
LOC105231737
LOC105231796
LOC105231892
LOC105231893
LOC105231900
LOC105231907
LOC105231943
LOC105231945
LOC105232076
LOC105232135
LOC105232211
LOC105232260
LOC105232264
LOC105232346
LOC105232349
LOC105232353
LOC105232361
LOC105232371
LOC105232398
LOC105232485
LOC105232539
LOC105232646
LOC105232648
LOC105232660
LOC105232761
LOC105232850

LOC105232851
LOC105232946
LOC105233001
LOC105233002
LOC105233050
LOC105233110
LOC105233137
LOC105233171
LOC105233193
LOC105233206
LOC105233254
LOC105233263
LOC105233269
LOC105233271
LOC105233317
LOC105233420
LOC105233474
LOC105233492
LOC105233506
LOC105233516
LOC105233537
LOC105233658
LOC105233767
LOC105233801
LOC105233873
LOC105233880
LOC105233908
LOC105233918
LOC105233926
LOC105233988
LOC105234063
LOC105234064
LOC105234075
LOC105234090
LOC105234107
LOC105234198
LOC105234227
LOC109579177
LOC109579215
LOC109579238
LOC109579347
LOC109579479

LOC109579480
LOC109579498
LOC109579524
LOC109579575
LOC109579590
LOC109579621
LOC109579628
LOC109579662
LOC109579763
LOC109579778
LOC109579859
LOC109579883
LOC109579897
LOC109579943
LOC109579965
LOC109580023
LOC109580030
LOC109580048
LOC109580083
LOC115065774
LOC115065990
LOC115066122
LOC115066154
LOC115066423
LOC115066590
LOC115066686
